# Epidemiological features of the 2024 pertussis outbreak in Gyeonggi Province, Korea

**DOI:** 10.4178/epih.e2025072

**Published:** 2025-12-13

**Authors:** Yeon Hwa Chang, Kyujin Chang, Yeong Jun Ju

**Affiliations:** 1 Infectious Disease Control Division, Health Bureau, Gyeonggi Provincial Government, Suwon, Korea; 2 Department of Public Health, Ajou University Graduate School of Public Health, Suwon, Korea; 3 Department of Preventive Medicine and Public Health, Ajou University School of Medicine, Suwon, Korea

**Keywords:** *Bordetella pertussis*, Pertussis vaccine, Hospitalization, Disease outbreaks

## Abstract

**OBJECTIVES:**

In 2024, Korea experienced a nationwide pertussis epidemic, with Gyeonggi Province accounting for nearly one-third of reported cases. This study investigated the epidemiological characteristics of the outbreak and explored the association between vaccination history and healthcare utilization.

**METHODS:**

We analyzed 14,275 pertussis cases reported in Gyeonggi Province in 2024 using de-identified national surveillance data. Comparisons were performed by age group (<20 vs. ≥20 years) and vaccination status (<20 years). The chi-square and Mann–Whitney *U* tests were used, and effect sizes were assessed using Cramér’s V.

**RESULTS:**

Of all cases, 89.8% occurred in individuals <20 years, particularly those aged 10-14 years. Children and adolescents were more often involved in clusters and had more identified contacts than adults, whereas adults had higher rates of hospitalization (13.2 vs. 5.9%) and emergency visits (4.4 vs. 0.9%; p<0.001). Among individuals <20 years, hospitalization was more common in the unvaccinated or unknown group (11.7%) than in the fully (5.9%) or partially vaccinated (5.5%) groups (p=0.045).

**CONCLUSIONS:**

The epidemic was concentrated in school-aged populations, particularly adolescents. While vaccination status showed a limited association with healthcare utilization, individuals who were unvaccinated or had an undocumented vaccination history experienced delayed diagnosis and higher care needs. These findings highlight the importance of strengthening adolescent-focused vaccination strategies and preparedness for future pertussis outbreaks.

## GRAPHICAL ABSTRACT

[Fig f1-epih-47-e2025072]

## Key Message

Analysis of the pertussis epidemic in Gyeonggi Province, home to one quarter of the national population, during the 2024 outbreak in Korea showed that cases were concentrated among school-aged adolescents. Individuals who were unvaccinated or had an undocumented vaccination history experienced delayed diagnosis and higher care needs. Improving adolescent- focused prevention strategies is essential to prevent pertussis re-emergence.

## INTRODUCTION

The Korea has maintained a high vaccination coverage of 90-95% through the National Immunization Program (NIP) over the past several decades, including mandatory vaccination against pertussis with the DTaP and Tdap vaccines [1-3]. Pertussis is a highly communicable respiratory disease transmitted via droplets, particularly affecting children and adolescents [4]. Owing to the vaccination program, cases of *Bordetella pertussis* infection have become rare in Korea, and pertussis-related hospitalizations have been infrequent. Most cases are characterized by mild to moderate symptoms, and vaccination remains the most effective strategy to prevent infection and disease progression [5,6]. According to the NIP, the Korean pertussis vaccination schedule includes DTaP, DTaP-IPV, or DTaP-IPV/Hib vaccines at 2 months, 4 months, and 6 months of age; a DTaP booster at 15-18 months; an additional DTaP or DTaP-IPV dose at 4-6 years; and a final booster with Tdap at 11-12 years [4].

The World Health Organization reported more than 151,000 cases of pertussis worldwide in 2018, which then declined before increasing again to more than 158,000 cases in 2023 [7]. In the United States, the number of cases increased by approximately 5-fold from 7,063 in 2023 to 35,435 in 2024, and in China, the number of cases reported in the first 3 months of 2024 increased by more than 10-fold compared to the same period in the previous year [8]. In 2023, Russia reported the highest number of cases at 52,783, while in 2024, China reported 476,690 cases, an increase of more than 11-fold (41,124 cases) compared to the previous year [7]. Vietnam also saw a 27-fold increase (from 40 cases) to 1,079 cases compared to the previous year, and Japan reported 4,095 cases of pertussis, a sharp increase over the preceding 2 years [7].

Between 2001 and 2023, a total of 3,231 pertussis cases were reported in Korea, corresponding to an annual incidence of just 0.02-1.00 per 100,000 population. However, in 2024, the Korea Disease Control and Prevention Agency (KDCA) reported a significant resurgence, with 48,048 cases, including 262 suspected cases, equivalent to approximately 92.9 per 100,000 population. Among them, 8,188 cases (17.0%) occurred in those aged 0-9 years and 35,134 cases (73.1%) were aged 10-19 years [9]. One infant death was also reported [9]. Notably, the number of severe cases—characterized by respiratory distress or failure, cyanosis, and even death—may increase proportionally as the total number of infections rises [5,6]. Such surges can place a substantial burden on individuals, communities, and the healthcare system by increasing the need for medical care, including hospitalizations [10,11].

Recent epidemiological studies from high-income countries have reported that even in settings with high vaccination coverage, the pertussis disease burden has shifted toward adolescents and adults, possibly due to waning immunity and changes in social contact patterns [12-14]. National surveillance data from Korea for 2010-2023 also suggest temporal trends driven by age, period, and cohort effects [15]. However, previous reports were primarily based on national aggregate trends and did not include a case-based, province-wide analysis during an epidemic period. Gyeonggi Province, which surrounds the capital city of Seoul, is the most populous region in the Korea, with 13.7 million residents (26.5% of the national population). In 2024, a total of 14,275 pertussis cases were reported in the province, accounting for 29.7% of all cases nationwide.

This study adds to the existing evidence by analyzing all reported pertussis cases in Gyeonggi Province during the large 2024 epidemic using individual-level surveillance data. We comprehensively assessed demographic factors, vaccination status, and healthcare utilization (hospitalizations and emergency department visits). Furthermore, a subgroup analysis was performed among children and adolescents with verifiable vaccination records in the NIP to provide detailed vaccination indicators that have not previously been reported. Therefore, this paper provides new empirical insights that complement and expand upon previous research and offers evidence to inform age-targeted vaccination strategies and strengthen public health preparedness [9].

## MATERIALS AND METHODS

### Data source and study population

Local public health centers in cities and districts conducted epidemiological investigations for every reported case, including medical record reviews, in accordance with the Infectious Disease Control and Prevention Act. The results were reported to the health authority through the Disinfection Information Management System, which operates under the National Notifiable Disease Surveillance System. The health authority provides de-identified data to researchers, excluding any individually identifiable information.

The authors obtained pertussis case data from Gyeonggi Province, Korea, for the period from January 1, 2024 to December 31, 2024. A total of 14,275 pertussis cases were included in the study, comprising 14,199 laboratory-confirmed cases and 76 suspected cases. All included cases were individuals diagnosed with pertussis or classified as suspected pertussis cases by healthcare providers and were residents of Gyeonggi Province. Cases reported by medical institutions that were later determined not to be pertussis were excluded from the analysis. Among the total cases, 12,816 children and adolescents whose vaccination history could be objectively verified were selected as the vaccination-related analysis group.

### Case definition and variables

The case definitions, classification standards, and diagnostic criteria were based on the 2024 KDCA Guidelines for the Management of Vaccine-Preventable Diseases and were applied uniformly across all provinces and municipalities nationwide.

A confirmed case was defined as a patient presenting with typical clinical symptoms of pertussis—such as coughing, paroxysmal cough, inspiratory whooping, or post-tussive vomiting—in whom Bordetella pertussis was isolated and identified from a respiratory specimen (e.g., nasopharyngeal swab or sputum) or who had a positive result on real-time polymerase chain reaction (PCR). A suspected case was defined as a patient who, despite having no laboratory confirmation or having tested negative, exhibited the above clinical symptoms and was clinically diagnosed by a physician based on epidemiological links, such as contact with a confirmed case or occurrence within a cluster in the same setting. For occurrence type, a sporadic case was defined as a single case with no identified epidemiological linkage, whereas a cluster-associated case was defined as 2 or more epidemiologically linked cases occurring within the same facility (e.g., school, hospital, workplace), excluding household clusters.

Contact tracing was conducted as a mandatory measure for all confirmed and suspected pertussis cases by local public health centers in each municipality. After a case was reported, the epidemiological investigation team at the local public health center conducted telephone interviews to identify family members and school or workplace contacts. If necessary, the epidemiological investigation team of the Gyeonggi Provincial Infectious Disease Control Division provided on-site investigation support. According to the guideline, a contact was defined as a close contact from the day of symptom onset until the end of isolation (or completion of 5 days of antibiotic treatment). The number of contacts was determined based on the final results recorded in each case’s epidemiological investigation report.

Diagnostic testing was primarily performed in medical facilities where patients received care, and results were reported to the public health authority. Laboratory reports included the test type (culture or real-time PCR) and the corresponding results. PCR testing generally targeted *B. pertussis*-specific genes, such as IS481, ptx, and ptxA, while some reports only indicated the presence or absence of toxin genes or other gene targets, reflecting variability in reporting systems. Antigen detection tests were not performed. Among all cases, 14,204 underwent real-time PCR testing and 25 underwent culture testing. Asymptomatic individuals with positive laboratory results (asymptomatic PCR-positive cases) were excluded from both confirmed and suspected case classifications because they did not reflect clinical illness or transmission risk.

The collected variables included sex, age, nationality, classification as a domestic or imported case, type of occurrence (sporadic or cluster-associated), laboratory test results, pertussis vaccination history, hospitalization (admission for treatment following pertussis diagnosis), emergency department visits (treatment sought in the emergency room due to pertussis), presence of underlying medical conditions (as self-reported by the patient), level of the reporting medical facility, interval from symptom onset to diagnosis, interval from diagnosis to notification, and number of contacts. Age-appropriate completion of pertussis vaccination was defined as receiving the number of vaccine doses required for one’s age according to the NIP schedule.

### Statistical analysis

Demographic and epidemiological characteristics were summarized using frequencies and percentages. For statistical comparisons, the chi-square test was applied to categorical variables, and continuous variables were compared using mean differences, with p-values of less than 0.05 considered to indicate statistical significance. Because the assumptions of normality and homogeneity of variance were not met, the non-parametric Mann–Whitney *U* test was used for comparisons of means.

Effect sizes for categorical variables were evaluated using Cramér’s V to provide additional context regarding the strength of associations. The interpretation of Cramér’s V followed Cohen’s (1988) conventional thresholds, with values of approximately 0.10, 0.30, and 0.50 indicating small, medium, and large associations, respectively. During the chi-square analyses, all expected cell counts were confirmed to be ≥5, thereby satisfying the test assumptions. Cramér’s V was subsequently reported as a complementary measure of effect size based on these verified conditions.

These analyses were performed to assess the association between age-appropriate completion of pertussis vaccination and clinical outcomes (hospitalization and emergency department visits). All statistical analyses were performed using SPSS version 29 (IBM Corp., Armonk, NY, USA).

### Ethics statement

The epidemiological investigation by local public health centers was conducted in accordance with the Korean Infectious Disease Control and Prevention Act. This study protocol was reviewed and granted exemption by the Public Institutional Review Board designated by the Korean Ministry of Health and Welfare (IRB No. P01-2025506-01-034).

## RESULTS

### Demographic and epidemiological characteristics

Among the 14,275 pertussis cases reported in Gyeonggi Province in 2024, 7,937 (55.6%) were male, with a mean age of 14.70±10.81 years, and 6,338 (44.4%) were female, with a mean age of 17.19±15.00 years. By age group, the largest proportion of cases occurred in those aged 10-14 years (7,443, 52.1%), followed by 15-19 years (2,894, 20.3%), 5-9 years (2,167, 15.2%), ≥20 years (1,459, 10.2%), and 0-4 years (312, 2.2%). The majority of patients (98.6%) were Korean nationals, and most infections (89.2%) were acquired domestically. Regarding the type of occurrence, 62.0% were sporadic cases, while 38.0% were associated with clusters. Laboratory testing was conducted for 99.7% of cases, and 99.5% of all reported cases were laboratory-confirmed.

In terms of vaccination history, 77.1% were fully vaccinated according to age-appropriate schedules, 13.7% were partially vaccinated, and 9.2% were either unvaccinated or had unknown vaccination status. With respect to healthcare utilization, 6.6% were hospitalized and 1.3% visited the emergency department. Underlying diseases were reported in 2.4% of patients, while 58.9% had no reported comorbidities and 38.8% had unknown comorbidity status. Regarding the type of reporting medical facility, 27.6% of patients were reported by hospitals or higher-level facilities and 72.4% by clinics. The mean interval from symptom onset to diagnosis was 4.13±12.01 days, and the mean interval from diagnosis to notification was 0.12±0.42 days. The average number of contacts per case was 15.17±32.48 (Table 1).

### Age-specific comparison of characteristics

Among the 12,816 individuals aged <20 years and 1,459 individuals aged ≥20 years, statistically significant differences were observed across most variables. Compared to adults, children and adolescents were more likely to be male, of Korean nationality, infected domestically, involved in cluster outbreaks, tested by laboratory methods, confirmed through laboratory results, and fully vaccinated according to their age-appropriate immunization schedule—all with statistical significance (p<0.001).

Although the absolute number of hospitalized patients was higher among those aged <20 years (751 cases, 5.9%) than among those aged ≥20 years (193 cases, 13.2%), the proportion was significantly lower in the younger group (p<0.001). Similarly, emergency department visits were more frequent in absolute number among the <20-year group (116 cases, 0.9%) than in the ≥20-year group (64 cases, 4.4%), but the proportion among the latter group was significantly greater (p<0.001). Significant differences were also observed in the presence of underlying diseases and in the type of reporting medical facility (p<0.001). The interval from symptom onset to diagnosis was longer in adults (p<0.001), whereas the number of identified contacts per case was higher among children and adolescents (p<0.001). A statistically significant difference in the mean number of contacts was also observed between the 10-19-year group (16.55±36.12) and the under-9 group (14.07±18.84) (analysis of variance, p=0.004). However, there was no statistically significant difference in the interval from diagnosis to notification (p=0.584) (Table 1).

### Vaccination history and healthcare utilization in children and adolescents

Among individuals younger than 20 years, characteristics were compared according to vaccination status: fully vaccinated, partially vaccinated, and unvaccinated or unknown. There was no statistically significant difference in vaccination status by sex (p=0.275). However, a significant difference was observed by nationality: Korean nationals accounted for 99.1% of the fully vaccinated group, 98.3% of the partially vaccinated group, and 64.9% of the unvaccinated or unknown group (p<0.001; Cramér’s V=0.265). Regarding the type of infection, cluster-associated cases accounted for 41.0% of the fully vaccinated group, 48.3% of the partially vaccinated group, and 30.9% of the unvaccinated or unknown group, showing a statistically significant difference (p<0.001; Cramér’s V=0.056). No significant differences were found in laboratory testing or confirmation status by vaccination group. For hospitalization, 5.9% of the fully vaccinated group and 5.5% of the partially vaccinated group were hospitalized, compared to 11.7% in the unvaccinated or unknown group, with a statistically significant difference (p=0.045; Cramér’s V=0.022). No significant difference was observed in emergency department visits (p=0.983). Statistically significant differences were found in the presence of underlying diseases by vaccination status (p<0.001; Cramér’s V=0.068), as well as in the type of reporting medical facility (p=0.011; Cramér’s V=0.049) (Table 2).

When the fully vaccinated and partially vaccinated individuals were grouped together and compared with those who were unvaccinated or had unknown vaccination status, no statistically significant differences were found in any of the variables listed in Table 2, including the Cramér’s V values. However, differences were observed in the 3 interval-related variables, as shown in Table 3. Specifically, the interval from symptom onset to diagnosis (p=0.021) and the number of contacts per case (p<0.001) showed statistically significant differences (Table 3).

## DISCUSSION

This study analyzed the epidemiological characteristics of the 2024 pertussis epidemic in Gyeonggi Province, Korea, and examined differences in vaccination history and healthcare utilization by age group. Among the total reported cases, 89.8% occurred in individuals under 20 years of age, highlighting a distinct age concentration of the outbreak. In this younger population, males were more frequently affected, whereas females predominated among adult cases. This pattern is consistent with reports from India, Taiwan, and Indonesia, where pertussis incidence has been highest among children and adolescents, suggesting that younger age groups continue to serve as the primary reservoir for transmission in many Asian regions with high vaccination coverage [12,13]. In contrast, some countries, such as China and France, have reported elevated incidence not only among children under 6 years old but also among adolescents and adults. These findings indicate that the age distribution of pertussis may vary across settings depending on national vaccination schedules, waning immunity, and underlying social contact structures [14].

In Korea, national surveillance data from 2009 to 2011 showed that individuals aged 11-20 years accounted for 26.3% of all pertussis cases [12]. In contrast, during the 2024 outbreak in Gyeonggi Province, this age group represented 72.4% of cases. This shift may be attributed to the higher average number of contacts among adolescents, which likely contributes to increased transmission risk. Although the number of contacts is rarely reported in the literature, this study found that children and adolescents had more identified contacts than adults. Moreover, cluster-related infections were significantly more common in those under 20 (41.9%) than in adults (3.7%). This finding aligns with previous research and underscores the heightened vulnerability of school-aged populations to infectious disease transmission in group settings [16,17]. It highlights the importance of strengthening infection prevention and control measures targeting children and adolescents.

Based on national surveillance data from 2010 to 2023 in Korea, pertussis cases were predominantly concentrated among children under 10 years of age (47.1%), whereas individuals aged 10-19 years accounted for only 25.6% of all reported cases [9]. In contrast, during the 2024 outbreak in Gyeonggi Province, the proportion of adolescents (10-19 years) increased markedly to 72.4%, while cases among children under 10 years decreased to 17.4%, indicating a shift in the predominant age group of infection from young children to adolescents. This age shift may be attributed to the higher average number of social contacts among adolescents, which increases opportunities for transmission in schools and other congregate settings. Although detailed contact data have been rarely reported in previous studies, the present analysis showed that children and adolescents had more identified contacts than adults. Moreover, a significant difference in the mean number of contacts was observed when using 20 years of age as the cutoff, and an additional significant difference was also identified between individuals aged 10-19 years and those under 10 years of age. The proportion of cluster-associated infections among individuals under 20 years (41.9%) was also substantially higher than that among adults (3.7%). Specifically, children under 9 years accounted for 21.2% of cluster-associated cases, whereas adolescents aged 10-19 years accounted for 46.9%, indicating that cluster outbreaks occurred more frequently among adolescents than among younger children. These findings suggest that adolescents may play a more central role in pertussis transmission than either younger children or adults. This underscores the heightened vulnerability of school-aged populations to pertussis transmission in group environments. Strengthening infection prevention and control strategies targeting adolescents should therefore be considered a public health priority.

Vaccination status was another key differentiator. Among individuals younger than 20 years, 84.9% had completed age-appropriate vaccination, whereas only 7.7% of adults were partially vaccinated and 83.4% had either incomplete or unknown vaccination history. In Korea, pertussis vaccination was widely introduced in the 1980s, and an electronic immunization registry system was established in 2002, with full implementation in 2015 [9]. Consequently, incomplete or undocumented vaccination is likely more common among adults younger than 40 years [15,18]. To minimize potential bias arising from such incomplete vaccination histories—which could lead to discrepancies in age-specific coverage and immunity levels—age restrictions were applied as part of the study design. Moreover, differences in vaccine formulations used over time may also have influenced immunity against *B. pertussis*. For example, the Td vaccine, which does not contain the pertussis antigen, remained in the national immunization schedule for adolescents until early 2025, and to avoid misclassification related to vaccine formulation, these doses were excluded from the vaccine effectiveness analysis as a design-based decision [4].

This study shows that adults had a higher prevalence of underlying medical conditions and were significantly more likely to be hospitalized or visit the emergency department than younger individuals. Although our dataset lacked detailed clinical information, such as specific diagnoses or severity classifications, previous studies conducted in New Zealand, the United States, and the United Kingdom have consistently demonstrated that comorbidities, including asthma and chronic obstructive pulmonary disease, increase the risk of complications and healthcare utilization among pertussis cases [19-21]. Therefore, it is plausible that the higher hospitalization and emergency care rates observed among adults in this study may result from a combination of these underlying health conditions, vaccination history, and waning immunity. Further investigations incorporating detailed clinical and immunological data are warranted to clarify the drivers of adult healthcare utilization during pertussis epidemics. Studies have shown that the effectiveness of the Tdap booster may decrease from 70-80% within 2 years of vaccination to less than 30% after 4-6 years [22]. This may explain the vulnerability observed in adolescents and adults across various high-income countries, including the United States, Australia, and Japan [12,23-26]. Accordingly, the increased risk of severe outcomes among adults may stem from both declining immunity and the presence of comorbidities, warranting integrated risk assessment in future research.

Among children and adolescents, those who were unvaccinated or had unknown vaccination history were more likely to be foreign nationals, and the location of infection was more often unknown rather than domestic. The association between nationality and vaccination status showed a moderate strength (Cramér’s V=0.265), likely reflecting Korea’s relatively high national vaccination coverage [27]. Although sporadic infection was more common than cluster infection in this group, the strength of this association was weak. Hospitalization was more frequent in unvaccinated or unknown-status individuals (11.7%) compared to those who were fully (5.9%) or partially vaccinated (5.5%). Although statistically significant, this association was also weak (Cramér’s V=0.022), indicating that vaccination status alone did not strongly predict hospitalization. Similarly, weak associations were observed for the type of occurrence and underlying disease status, whereas nationality showed a moderate correlation with vaccination status (Cramér’s V=0.265). These findings suggest that while vaccination remains a key determinant in pertussis prevention, other contextual and social factors—such as nationality, health-seeking behavior, and documentation practices—may play a substantial role in shaping outbreak patterns. No significant difference was observed in emergency department visits by vaccination status. Importantly, the interval from symptom onset to diagnosis was significantly longer in the unvaccinated or unknown group, suggesting that this population may be more vulnerable to delayed recognition and care. Paradoxically, this group also had fewer reported contacts, which may reflect differences in health-seeking behavior, socioeconomic factors, or documentation practices. Further investigation is needed to understand these patterns.

This study has several limitations. First, detailed clinical information, such as disease severity, specific diagnoses, and length of hospital stay, was not available, limiting the ability to capture the full spectrum of clinical presentations. Second, potential risk modifiers—including socioeconomic status, school environment, and hygiene practices—were not assessed, restricting a comprehensive understanding of infection risk factors. Third, serologic and cellular immune response data were not collected. Fourth, pathogen genotyping and pertussis toxin gene testing were not performed, precluding analysis at the molecular level. Despite these limitations, this study represents the first and largest comprehensive analysis of a regional pertussis epidemic in Korea, providing important methodological and public health insights. Notably, this study quantified the number of contacts per case, which has been underreported in prior research, and presented effect sizes (Cramér’s V) to evaluate the strength of association between categorical variables. By doing so, it moves beyond simple statistical significance testing and enables a more nuanced interpretation of the magnitude and direction of associations. Furthermore, a subgroup analysis of individuals aged under 20 years—whose vaccination histories could be verified through the NIP registry—was conducted to examine the linkage between vaccination status, healthcare utilization (hospitalization and emergency department visits), and diagnostic delay. The study also explicitly excluded Td vaccines lacking pertussis antigen from vaccine effectiveness analyses to minimize misclassification bias arising from historical differences in vaccine formulations.

These findings collectively suggest that individuals who are unvaccinated or have an unknown vaccination history are more vulnerable to diagnostic delay and disease burden, underscoring the need for targeted strategie, such as strengthening infection prevention and control measures in school-based settings and improving vaccination accessibility among immigrants and undocumented populations [4,9,27]. In addition, this study provides empirical evidence for identifying high-risk groups based on vaccination history and formulating targeted booster vaccination strategies. The higher prevalence of comorbidities and greater healthcare utilization observed among adults are consistent with cohort studies from New Zealand, the United States, and the United Kingdom, which have reported similar associations between pertussis-related complications and underlying conditions such as asthma and chronic obstructive pulmonary disease [19-21]. These findings support the need for booster vaccination policies and integrated risk assessment frameworks for older adults and individuals with chronic diseases in Korea [12,23-26].

In summary, this study provides the first population-based, large-scale epidemiologic analysis of a pertussis epidemic in Korea, particularly in Gyeonggi Province. It offers actionable evidence for identifying vulnerable populations and advancing age-based and risk-based vaccination policies, thereby contributing to the strengthening of national public health preparedness and pertussis control strategies.

## Figures and Tables

**Figure f1-epih-47-e2025072:**
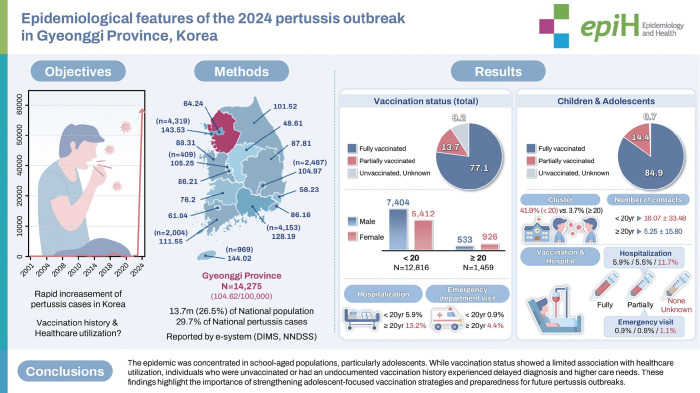


**Table 1.** General characteristics of pertussis cases and comparison between individuals aged <20 years and ≥20 years

Characteristics	Total (n=14,275)	Age (yr)		p-value^1^
		<20 (n=12,816)	≥20 (n=1,459)	
Sex				<0.001
Male	7,937 (55.6)	7,404 (57.8)	533 (36.5)	
Female	6,338 (44.4)	5,412 (42.2)	926 (63.5)	
Nationality				<0.001
Korea	14,072 (98.6)	12,658 (98.8)	1,414 (96.9)	
Other	203 (1.4)	158 (1.2)	45 (3.1)	
Country of infection				<0.001
Korea/domestic	12,727 (89.2)	11,486 (89.6)	1,241 (85.1)	
Imported	34 (0.2)	31 (0.2)	3 (0.2)	
Unknown	1,514 (10.6)	1,299 (10.1)	215 (14.7)	
Type of occurrence				<0.001
Cluster	5,430 (38.0)	5,376 (41.9)	54 (3.7)	
Sporadic	8,845 (62.0)	7,440 (58.1)	1,405 (96.3)	
Laboratory test performed				<0.001
Yes	14,229 (99.7)	12,782 (99.7)	1,441 (98.8)	
No	46 (0.3)	34 (0.3)	18 (1.2)	
Diagnosis				<0.001
Confirmed	14,199 (99.5)	12,764 (99.6)	1,435 (98.4)	
Suspected	76 (0.5)	52 (0.4)	24 (1.6)	
Vaccination status				<0.001
Fully vaccinated (age-appropriate)	11,007 (77.1)	10,877 (84.9)	130 (8.9)	
Partially vaccinated	1,957 (13.7)	1,845 (14.4)	112 (7.7)	
Unvaccinated/unknown	1,311 (9.2)	94 (0.7)	1,217 (83.4)	
Hospitalization				<0.001
Yes	944 (6.6)	751 (5.9)	193 (13.2)	
No	13,331 (93.4)	12,065 (94.1)	1,266 (86.8)	
Emergency department visit				<0.001
Yes	180 (1.3)	116 (0.9)	64 (4.4)	
No	14,095 (98.7)	12,700 (99.1)	1,395 (95.6)	
Underlying disease				
Present	336 (2.4)	217 (1.7)	119 (8.2)	
Absent	8,406 (58.9)	7,509 (58.6)	897 (61.5)	
Unknown	5,533 (38.8)	5,090 (39.7)	443 (30.4)	<0.001
Type of reporting facility				<0.001
Hospital or higher	3,940 (27.6)	3,413 (26.6)	527 (36.1)	
Clinic	10,335 (72.4)	9,403 (73.4)	932 (63.9)	
Interval (day)				<0.001^2^
Symptom onset to diagnosis	4.13±12.01	3.95±12.35	5.69±8.30	
Diagnosis to notification	0.12±0.42	0.12±0.43	0.11±0.40	0.584^2^
No. of contacts (n=13,453)	15.17±32.48	16.07±33.48	5.52±15.80	<0.001

Values are presented as number (%) or mean±standard deviation.

1 Chi-square test.

2 Mann–Whitney *U* test.

**Table 2.** Vaccination status and characteristics of individuals aged <20 years (n=12,816)

Variables	Fully vaccinated	Partially vaccinated	Unvaccinated/unknown	p-value^1^	Cramér’s V
Sex				0.275	0.014
Male	6,252 (57.5)	1,095 (59.3)	57 (60.6)		
Female	4,625 (42.5)	750 (40.7)	37 (39.4)		
Nationality				<0.001	0.265
Korea	10,783 (99.1)	1,814 (98.3)	61 (64.9)		
Other	94 (0.9)	31 (1.7)	33 (35.1)		
Country of infection				0.044	0.020
Korea/domestic	9,745 (89.6)	1,665 (90.2)	76 (80.9)		
Imported	28 (0.3)	3 (0.2)	0 (0)		
Unknown	1,104 (10.1)	177 (9.6)	18 (19.1)		
Type of occurrence				<0.001	0.056
Cluster	4,456 (41.0)	891 (48.3)	29 (30.9)		
Sporadic	6,421 (59.0)	954 (51.7)	65 (69.1)		
Laboratory test performed				0.268	0.014
Yes	10,850 (99.8)	1,839 (99.7)	93 (98.9)		
No	27 (0.2)	6 (0.3)	1 (1.1)		
Diagnosis				0.595	0.009
Confirmed	10,833 (99.6)	1,838 (99.6)	93 (98.9)		
Suspected	44 (0.4)	7 (0.4)	1 (1.9)		
Hospitalization				0.045	0.022
Yes	638 (5.9)	102 (5.5)	11 (11.7)		
No	10,239 (94.1)	1,743 (94.5)	83 (88.3)		
Emergency department visit				0.983	0.002
Yes	98 (0.9)	17 (0.9)	1 (1.1)		
No	10,779 (99.1)	1,828 (99.1)	93 (98.9)		
Underlying disease				<0.001	0.068
Present	194 (1.8)	19 (1.0)	4 (4.3)		
Absent	6,570 (60.4)	886 (48.0)	53 (56.4)		
Unknown	4,113 (37.8)	940 (50.9)	37 (39.4)		
Type of reporting facility				<0.001	0.049
Hospital or higher	2,855 (26.2)	510 (27.6)	48 (51.1)		
Clinic	8,022 (73.8)	1,335 (72.4)	46 (48.9)		

Values are presented as number (%).

1 Chi-square trend test.

**Table 3.** Comparison of vaccination status and characteristics among individuals aged <20 years (n=12,816)

Variables	Interval (day)			p-value^1^	Interval (day)		p-value^2^
	Fully vaccinated	Partially vaccinated	Unvaccinated/unknown		Fully or partially vaccinated	Unvaccinated/ unknown	
Symptom onset to diagnosis	3.91±12.82	4.07±9.20	6.33±9.85	0.151	3.93±12.36	6.33±9.85	0.021
Diagnosis to notification	0.12±0.42	0.13±0.46	0.10±0.33	0.559	0.12±0.43	0.10±0.33	0.563
No. of contacts	15.92±33.14	17.23±36.03	10.33±13.26	0.079	16.11±33.58	10.33±13.26	<0.001

Values are presented as mean±standard deviation.

1 Analysis of variance.

2 *t*-test.
